# Isoform‐specific upregulation of FynT kinase expression is associated with tauopathy and glial activation in Alzheimer's disease and Lewy body dementias

**DOI:** 10.1111/bpa.12917

**Published:** 2021-01-29

**Authors:** Clara Y. B. Low, Jasinda H. Lee, Frances T. W. Lim, Chingli Lee, Clive Ballard, Paul T. Francis, Mitchell K. P. Lai, Michelle G. K. Tan

**Affiliations:** ^1^ Department of Clinical Translational Research Singapore General Hospital Outram Singapore; ^2^ Department of Pharmacology Yong Loo Lin School of Medicine Kent Ridge Singapore; ^3^ Institute for Health Research University of Exeter Medical School Exeter UK; ^4^ Wolfson Centre for Age‐Related Diseases King's College London London UK

**Keywords:** alternative splicing, Alzheimer's disease, Fyn kinase, glial activation, Lewy body dementia, tauopathy

## Abstract

Cumulative data suggest the involvement of Fyn tyrosine kinase in Alzheimer's disease (AD). Previously, our group has shown increased immunoreactivities of the FynT isoform in AD neocortex (with no change in the alternatively spliced FynB isoform) which associated with neurofibrillary degeneration and reactive astrogliosis. Since both the aforementioned neuropathological features are also variably found in Lewy Body dementias (LBD), we investigated potential perturbations of Fyn expression in the post‐mortem neocortex of patients with AD, as well as those diagnosed as having one of the two main subgroups of LBD: Parkinson's disease dementia (PDD) and dementia with Lewy bodies (DLB). We found selective upregulation of FynT expression in AD, PDD, and DLB which also correlated with cognitive impairment. Furthermore, increased FynT expression correlated with hallmark neuropathological lesions, soluble β‐amyloid, and phosphorylated tau, as well as markers of microglia and astrocyte activation. In line with the human post‐mortem studies, cortical FynT expression in aged mice transgenic for human P301S tau was upregulated and further correlated with accumulation of aggregated phosphorylated tau as well as with microglial and astrocytic markers. Our findings provide further evidence for the involvement of FynT in neurodegenerative dementias, likely via effects on tauopathy and neuroinflammation.

## Graphical abstract

Splice variant‐specific upregulation of FynT kinase correlated with clinical and neuropathological markers of Alzheimer's Disease (AD) and Lewy body Dementias (LBD). Our results suggest that FynT should be further evaluated as a novel therapeutic target in AD and LBD.

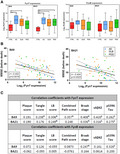

**Abbreviations**
AD: Alzheimer's disease
ANOVA: analysis of variance
AP: amyloid plaques
BA: Brodmann area
BA21: mid temporal gyrus
BA9: dorsolateral and medial prefrontal cortex
CDK5: cyclin‐dependent kinase 5
DLB: dementia with Lewy bodies
FDR: false discovery rate
FynB: Fyn kinase isoform B
FynT: Fyn kinase isoform T
GO: gene ontology
GSK3: glycogen synthase kinase 3
HTA: Human Transcriptome Array
HTRF: Homogenous Time‐Resolved Fluorescence
LB: Lewy bodies
LBD: Lewy body dementias
MMSE: Mini‐Mental State Examination
NFT: neurofibrillary tangles
PDD: Parkinson's disease dementia
PMI: post‐mortem interval
RIN: RNA Integrity Number
RT‐PCR: reverse transcription polymerase chain reaction
sAβ42: soluble fraction of β‐amyloid_1‐42_ peptide

## 1 INTRODUCTION

Alzheimer's disease (AD) and Lewy body dementias (LBD) are respectively the number one and two causes of neurodegenerative dementia in the elderly, and contribute to significant morbidity for both sufferers and their caregivers, as well as to global health‐care burden (1, 2, 3). LBD consists of two clinical subtypes: Parkinson's disease dementia (PDD) and dementia with Lewy bodies (DLB), where differentiation is mainly based on the “one‐year rule” in which patients presenting with dementia before, or within a year of, Parkinsonism symptoms are diagnosed as DLB, whereas dementia occurring >1 year after Parkinsonism is considered to be PDD (4). While PDD and DLB share similar neuropathological features, especially the presence of cortical aggregated α‐synuclein‐containing Lewy bodies (LB), significant differences in clinical presentation as well as neurochemical perturbations have been reported between them (5, 6, 7, 8, 9, 10), giving rise to ongoing debate on whether PDD and DLB are separate entities or part of the same spectrum of LBD (11, 12, 13, 14). Interestingly, in addition to LB, both PDD and DLB manifest variable burdens of hallmark AD lesions, namely intercellular amyloid plaques (AP) consisting mainly of insoluble, aggregated β‐amyloid peptides (Aβ); and intracellular neurofibrillary tangles (NFT) formed from aggregation of hyperphosphorylated tau proteins into paired helical filaments (15, 16, 17, 18). Furthermore, the presence of chronic neuroinflammation and glial activation, which are intimately linked to both AP and NFT (19, 20), are also salient features of AD and LBD (21, 22). Therefore, studies focused on the pathophysiological mechanisms and effects of AP and NFT formation (23) should consider the applicability and implications of their findings to LBD.

Given the diverse biological functions (ranging from regulating brain function to modulating T‐cell signaling) attributed to Fyn, a member of the Src family tyrosine kinases (24), it is perhaps unsurprising that Fyn kinase has been implicated in AD pathophysiology (25). As Fyn is alternatively spliced into two major isoforms: brain‐predominant FynB and immune cells‐predominant FynT, we have measured FynB versus FynT immunoreactivities in post‐mortem AD neocortex, and found selective increases in FynT which also associated with neurofibrillary degeneration and reactive astrogliosis (26). These findings are in line with *in vitro* data showing that prolonged inflammatory response and activation in astrocytes are mediated by pro‐inflammatory cytokine‐induced FynT (27). However, the status of Fyn isoform expression as well as potential associations with neuropathological features and neuroinflammatory markers in LBD is currently unknown. In this study, we identified differential splicing of Fyn using a human transcriptome microarray approach, and further confirmed our findings with real‐time RT‐PCR of Fyn isoform expression in neocortex of AD and LBD patients together with aged non‐demented subjects, and correlated the measures with Aβ and phospho‐Tau concentrations, neuroinflammatory markers, neuropathological and clinical scores. To further study possible links between Fyn perturbations and NFT formation, we also monitored longitudinal changes of Fyn isoform expression in association with tau pathology and neuroinflammation in a transgenic mouse model of human tauopathy which harbors the human P301S tau mutation (28).

## 2 METHODS

### 2.1 Patients, clinical and neuropathological assessments

Post‐mortem brain tissues from the frontal (Brodmann Area, BA9, dorsolateral/medial prefrontal cortex) and temporal (BA21: middle temporal gyrus) regions of subjects with AD, DLB, PDD, as well as elderly controls (CTRL) without neurological or psychiatric diseases were obtained from the University Hospital Stavanger, Newcastle Brain Tissue Resource, the London Neurodegenerative Diseases Brain Bank and the Thomas Willis Brain Collections at Oxford University, the UK sites being part of the Brains for Dementia Research network (29). Subjects with dementia were part of longitudinal studies and were followed up annually with clinical assessments including the Mini‐Mental State Examination (MMSE) (30) to measure cognitive decline until death, at which time informed consent was obtained from next‐of‐kin before removal of brains. Consensus criteria used for clinical diagnoses of AD, DLB, and PDD with neuropathologic confirmation have been previously described in detail (31). In addition, semiquantitative scoring (0 = None, 1 = Sparse, 2 = Moderate, and 3 = Abundant) of AP, NFT/neuropil threads, and LB/Lewy neurites as visualized by immunostaining with 4G8, AT8, and α‐synuclein antibodies, respectively, were performed by neuropathologists blinded to clinical diagnosis as previously described (18). For the neurochemical and gene expression studies, 1 cm^3^ frozen brain chunks were obtained from BA9 and BA21 of the hemisphere contralateral to the one used for neuropathologic studies, then, stored at −80°C before further processing for homogenate preparation and RNA isolation.

### 2.2 Brain tissues from transgenic mice

The P301S tau transgenic mouse line PS19 (B6 N.Cg‐Tg (Prnp‐MAPT*P301S)PS19Vle/J) were obtained from the Jackson Laboratory (Bar Harbor, ME, USA) and bred with wild‐type (WT) control, C57BL/6 NJ to generate hemizygotes and WT littermate controls. The strain harbors the 1N4R isoform of human MAPT gene with the P301S mutation, driven by mouse prion protein promoter which expressed the human P301S tau at levels fivefold higher than endogenous mouse tau (28). Brains from both genotypes were harvested directly after CO_2_ euthanasia before 6 months (mo) old (WT: n = 13, 8 M/5F, 4.9 ± 0.2 mo; P301S: n = 13, 6 M/7F, 4.7 ± 0.2 mo) and after 6 months old (WT: n = 18, 10 M/8F, 8.6 ± 0.4 mo; P301S: n = 18, 9 M/9F, 8.9 ± 0.4 mo). After removing cerebellum, olfactory bulb, and meninges, the cortex were separated into left and right hemispheres for RNA isolation and brain homogenate preparation, respectively. All experimental procedures conducted in the study were approved by the SingHealth Institutional Animal Care and Use Committee and carried out in accordance with the approved guidelines and regulations.

### 2.3 Brain tissue processing

Unless otherwise specified, all chemicals and reagents were purchased from Sigma Aldrich (St Louis, MO, USA) and of analytical grade. Frozen human brain chunks from BA9 and BA21 were thawed on ice, dissected free of meninges and white matter, then, homogenized (21) with an Ultra‐Turrax® T‐25 homogenizer (IKA‐Werke, Staufen, Germany) in ice‐cold buffer (50 mM Tris‐HCl, 120 mM NaCl, 5 mM KCl, and pH 7.4) with cOmplete™ protease inhibitor cocktail and PhosSTOP™ phosphatase inhibitor tablets (Roche Life Science, Penzberg, Germany). Brain homogenates from mice were prepared as above without the prior dissection step. For both human and mice brains, representative portions were kept in TRIzol® reagent (Invitrogen Inc., Carlsbad, CA, USA) at −80°C for subsequent RNA extraction.

### 2.4 Enzyme‐linked immunosorbent assays (ELISA) of pS396 Tau and soluble Aβ42 in human neocortex

Aliquots of brain homogenates was treated with 5 M Guanidine for detection of tau with serine phosphorylation at position 396 (pS396 Tau) by ELISA according to manufacturer's instructions (KHB7031, Thermo Fisher Scientific, Waltham, MA, USA). Separate homogenate aliquots were agitated using a sonicator for 30 mins at 4°C before centrifugation at 6000 g, 30 min at 4°C, with the supernatant used for detection of soluble Aβ_1‐42_ peptides (sAβ42) by ELISA according to manufacturer's instructions (KHB3441, Thermo Fisher Scientific). Concentrations values in pg/mL were normalized to protein content of each sample (determined using Pierce Coomassie Plus Reagent, Thermo Fisher Scientific) and presented as pg/mg protein.

### 2.5 RNA isolation, reverse transcription polymerase chain reaction (RT‐PCR), and capillary electrophoresis

Brain tissue kept frozen in TRIzol® reagent (Invitrogen Inc., Carlsbad, CA, USA) were thawed and processed for RNA extraction following manufacturer's instructions. A Fragment Analyzer™ system (Advanced Analytical Technologies Inc, Ankeny, IA, USA) was used for evaluating RNA quality, with RIN <3.0 excluded from further analysis. 2 μg of RNA was reverse‐transcribed to cDNA using High‐Capacity cDNA RT kit (Applied Biosystems, Foster City, CA, USA) in accordance to manufacturer's protocol. Semiquantitative measurements of gene expression were performed on the 7500 Fast Real‐time PCR system (Applied Biosystems) or CFX96 ^TM^ Real‐time PCR system (BioRad) using GoTaq® qPCR Master Mix (Promega, Madison, WI, USA). The primer sequences used in this study are listed in Table S1. All real‐time RT‐PCR assays were performed in duplicate. Standard curves of each gene were generated independently by 10x serial dilution of template DNA. The relative signal intensity of each sample was calculated according to the corresponding standard curve. Normalization was performed in each sample by dividing the relative signal intensity of gene of interest to geometric mean of β‐actin, GAPDH, and 18S rRNA. To determine FynT to FynB ratios using capillary electrophoresis, a pair of common primers spanning the alternative spliced exon of Fyn were used as described previously (26). Peak areas of FynT (219 bp DNA amplicon) and FynB (228 bp DNA amplicon) in each electropherogram were translated into expression level and used to determine ratios of FynT to FynB expression.

### 2.6 Human Transcriptome Array (HTA) data and Gene Ontology (GO) term enrichment analyses

A subset of samples (9 CTRL, 9 AD, 12 DLB, and 12 PDD) from BA9 were processed for high‐throughput transcriptome profiling using the GeneChip® HTA 2.0 array (Affymetrix, Santa Clara, CA, USA). The data set has been deposited with the Gene Expression Omnibus (GEO) (Access #GSE150696). Using the Gene View module of Partek Genomics Suite® 7.0 software (Partek Inc, St Louis, MO, USA), we were able to allocate differentially expressed probesets to specific transcript variants of Fyn. Expressed probesets which were significantly correlated with the FynT‐specific probesets (PSR06025242.hg.1) at false discovery rate (FDR)(32) cutoff of 5% were retrieved. Genes with ≥10 representative probesets that consistently correlated with FynT were subject to Gene Ontology (GO) term enrichment analyses using DAVID Bioinformatics Resources 6.7 (http://david.abcc.ncifcrf.gov/) (33, 34), with Benjamini's enrichment *p* values adjustment for multiple testing using FDR of 5%. For this study, we focused on GO categories of Biological Processing and Cellular components, after removing general GO terms with enrichment factor <2.

### 2.7 Homogenous Time‐Resolved Fluorescence (HTRF) assays of mouse brain homogenates

Cisbio® HTRF assays (PerkinElmer Inc. Waltham, MA, USA), a technology combining standard fluorescence resonance energy transfer (FRET) with time‐resolved measurement of fluorescence, were used for measuring total human tau protein (MAP‐Tau Kit), phosphorylated tau (Phospho‐Ser202/Thr205 human TAU kit), and aggregated tau (Tau aggregation kit) according to manufacturer's instructions. A portion of the right hemisphere was homogenized in Cisbio lysis buffer with blocking reagent using 5 mm stainless steel beads in a TissueLyser LT (Qiagen, Venlo, the Netherlands) tissue disruptor (two rounds of 50 Hz, 2 min). After centrifugation at 21,000 g for 20 min at 4°C, supernatants were measured for protein concentration using the Bradford protein assay kit (BioRad, Hercules, CA, USA). Equal amounts of brain homogenate were then diluted and incubated with respective antibodies for 24 h at room temperature. Samples were excited at 340 nm and emission values collected at 620 nm (Donor) and 665 nm (Acceptor) using EnSpire® multimode plate reader (Perkin Elmer). For data analysis, HTRF ratio was calculated for each individual well by the ratio of the two emission values (Signal 665 nm/Signal 620 nm) and multiplied by 10,000 to ease data processing. Data were presented as Delta F% which reflected the signal to background of the assay, and calculated as [HTRF ratio (sample) ‐ HTRF ratio (negative control)]/HTRF ratio (negative control) x 100.

### 2.8 Statistical analyses

Pairwise differences among groups were analyzed by Kruskal–Wallis analysis of variance (ANOVA) with *post hoc* Dunn's test or data transformed to log_2_ base value and analyzed using one‐way ANOVA with Bonferroni's *post hoc* tests. Two‐way ANOVA with Bonferroni's *post hoc* tests were applied to transgenic mice study with genotype and age as independent variables. Pearson's or Spearman's correlations was used to for parametric or nonparametric variables, respectively. For all analyses, two‐tailed *p* values of <0.05 were considered to be statistically significant. Statistical analyses and plotting of graphs were performed using the PRISM Version 5 software (GraphPad, San Diego, CA, USA).

## 3 RESULTS

### 3.1 Demographic and disease variables of the study cohort

Table 1 shows the maximum available numbers of aged controls (CTRL) as well as a community‐based cohort of PDD, DLB, and AD patients. Groups were not significantly different in age at death and post‐mortem interval (Kruskal–Wallis ANOVA *p* > 0.05). Within the dementia subgroups (AD, PDD, and DLB), no significant difference in dementia severity (indicated by MMSE decline per year) was observed (Kruskal–Wallis ANOVA *p* > 0.05). Braak staging (35) for extent of AD pathological changes showed all but one of the aged CTRL in Braak stage ≤II, with one in III–IV (another four CTRL did not have Braak staging data). In contrast, 10 out of 13 available AD cases were Braak V/VI, with the rest in Braak III/IV. PDD and DLB showed variable AD pathology ranging from Braak ≤II to V/VI, with DLB showing higher numbers of those with more advanced stages compared to PDD. Furthermore, ELISA measures of sAβ42 and pS396Tau, used respectively as markers of amyloid and neurofibrillary tangle burden, were also raised in the dementia subgroups, with the highest levels in AD, and the lowest in PDD (Table 1). These neuropathological and biochemical features are in line with previous clinical and post‐mortem observations of AD and Lewy body dementias, especially with regards to PDD harboring the lowest AD pathological burden among the dementia subgroups (17, 18).

**TABLE 1:** Demographic and disease variables of a cohort of patients with neuropathologically confirmed AD and LBD

	CTRL	AD	DLB	PDD
	(n = 16)	(n = 13)	(n = 39)	(n = 27)
*Demographics* ^a^				
Gender (male/female)	9 M/7F	5 M/8F	26 M/13F	15 M/12F
Age at death (years)	82.0 ± 1.7	86.1 ± 1.9	80.9 ± 1.1	79.3 ± 1.2
Postmortem interval (hours)	45.7 ± 5.9	36.7 ± 7.1	43.7 ± 4.9	33.5 ± 3.0
MMSE before death	–	9 ± 2.1 (n = 13)	14 ± 1.5 (n = 32)	12 ± 1.6 (n = 25)
Braak staging				
0–II	11 (91.7%)	0 (0%)	6 (15.4%)	18 (66.7%)
III–IV	1 (8.3%)	3 (23%)	20 (51.3%)	7 (26%)
V–VI	0 (0%)	10 (77%)	13 (33.3%)	2 (7.3%)
*ELISA assay*				
Soluble Aβ42 (pg/mg) (BA9)	5.6 ± 1.5 (n = 15)	33.2 ± 2.2* (n = 13)	29.6 ± 2.4* (n = 37)	10.9 ± 1.8**, *** (n = 26)
Soluble Aβ42 (pg/mg) (BA21)	6.2 ± 1.5 (n = 15)	27.5 ± 2.4* (n = 13)	25.2 ± 1.9* (n = 39)	10.3 ± 1.8**, *** (n = 26)
pS396Tau (pg/mg) (BA9)	5.4 ± 2.7 (n = 16)	168.4 ± 33.3* (n = 13)	47.6 ± 13.8** (n = 39)	2.2 ± 0.6**, *** (n = 27)
pS396Tau (pg/mg) (BA21)	25.1 ± 5.6 (n = 15)	524.8 ± 92.9* (n = 13)	215.1 ± 48** (n = 38)	61.9 ± 25.6** (n = 26)

Data are presented as mean ± S.E.M.

^a^ Maximum available n. Not all samples were available for all assessments. Available n listed in individual assessments.

* Significantly different compared to CTRL (Kruskal–Wallis ANOVA with *post hoc* Dunn's test, *p* < 0.05).

** Significantly different compared to AD (Kruskal–Wallis AONVA with *post hoc* Dunn's test, *p* < 0.05).

*** Significantly different compared to DLB (Kruskal–Wallis ANOVA with *post hoc* Dunn's test, *p* < 0.05).

### 3.2 Selective upregulation of FynT isoform expression in neocortex of AD and LBD

**FIGURE 1:**
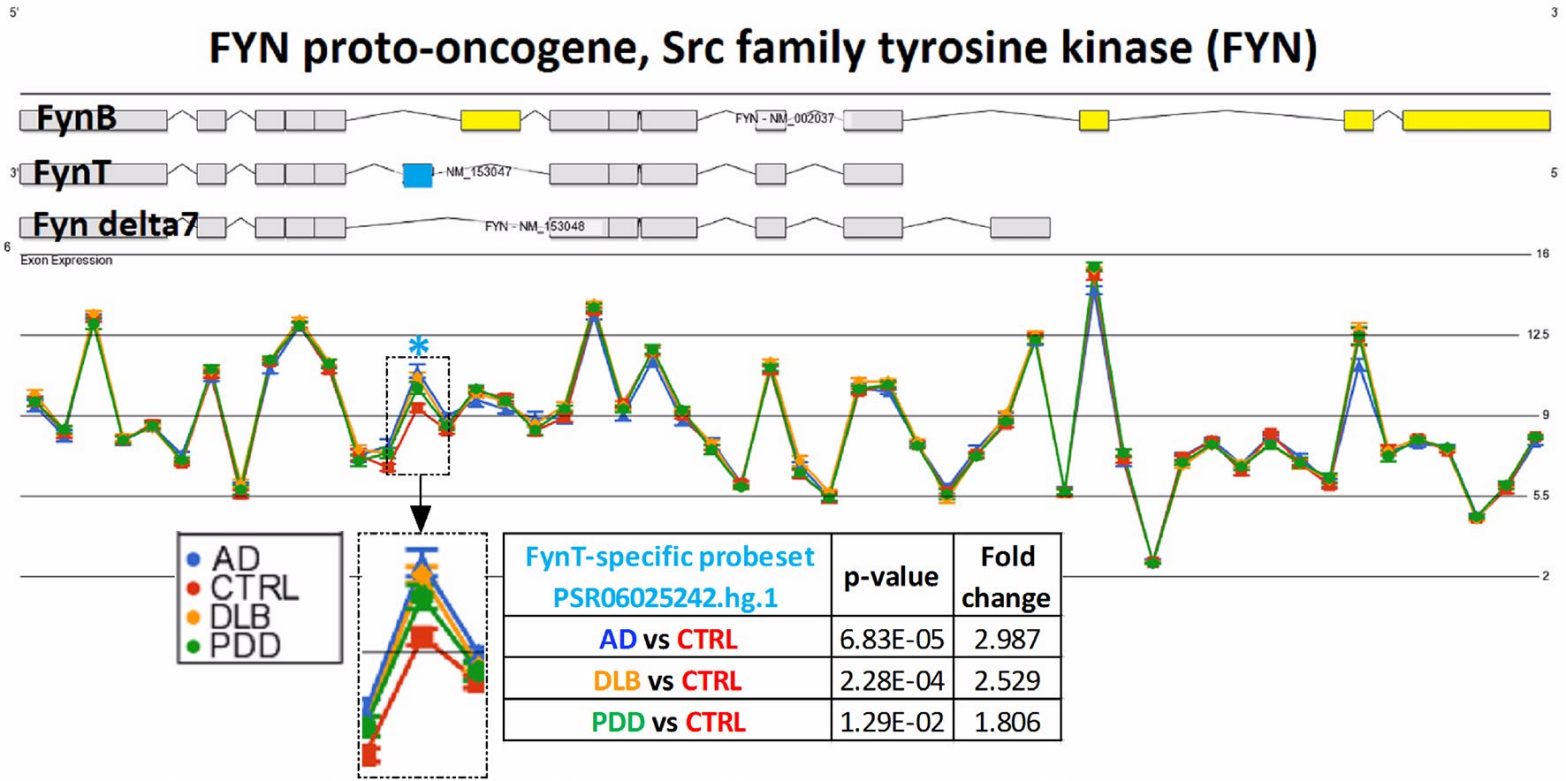
Specific upregulation of FynT isoform expression in prefrontal cortex of AD and LBD. Gene View plot demonstrates differential alternative splicing of Fyn in BA9 samples of CTRL (n = 9, red), AD (n = 9, blue), DLB (n = 12, amber), and PDD (n = 12, green), from data derived from Affymetrix HTA array. Light blue asterisk indicated the PSR06025242.hg.1 probeset, with a magnified view shown in the inset, corresponding to the FynT‐specific exon highlighted in blue. FynB‐specific exons are highlighted in yellow. The Y‐axis is in Log_2_ scale intensity value. The *p* values shown are of Alt‐splicing ANOVAs using the Partek Genomic Suite® software

**FIGURE 2:**
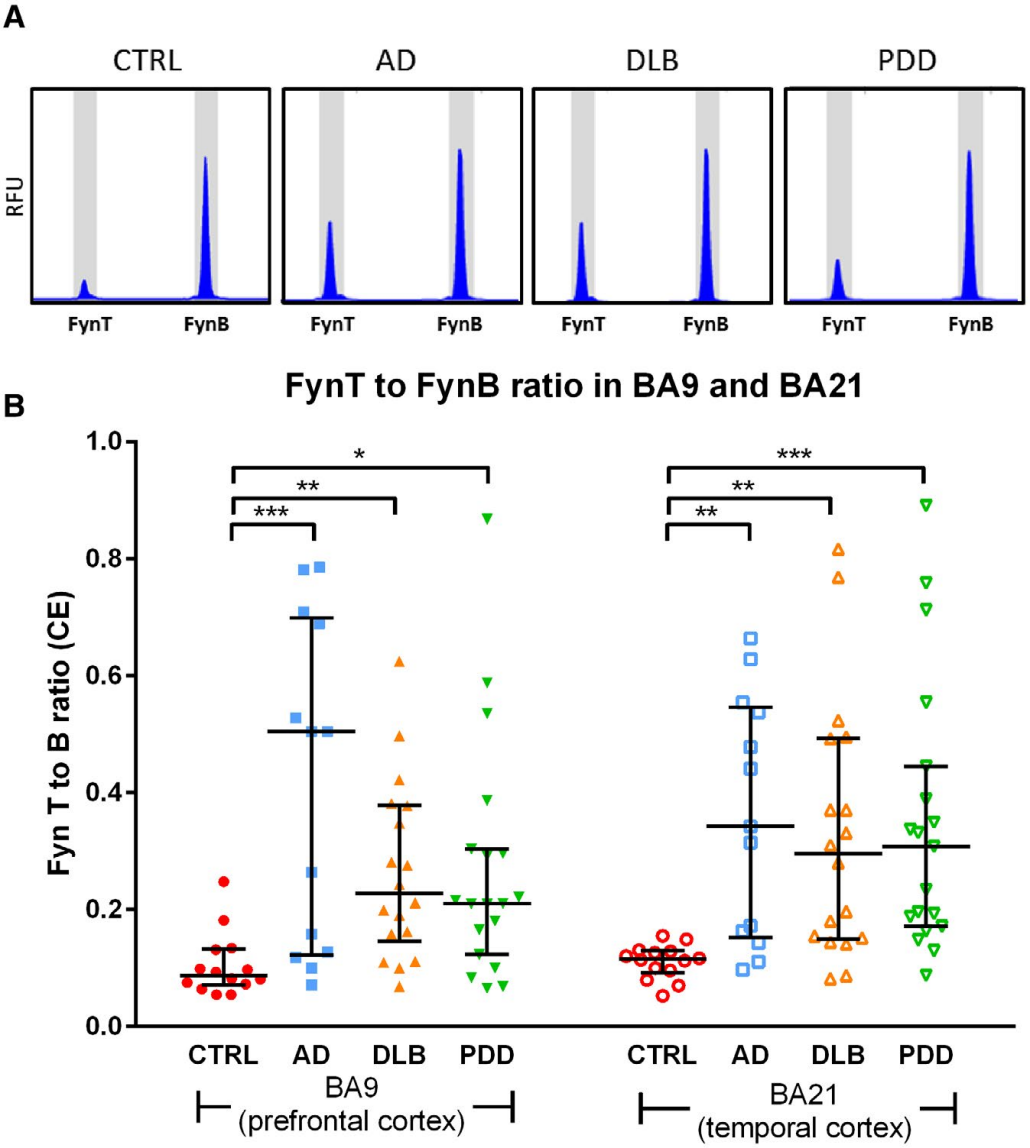
Increased FynT to FynB ratios in prefrontal and temporal cortex of AD and LBD. (A) Representative electropherograms of the proportional levels of FynB and FynT in each sample as determined by RT‐PCR using common primers spanning the alternatively spliced exon of Fyn followed by capillary electrophoresis. Relative fluorescence units (RFU) of FynT peak (219 bp DNA amplicon) and FynB peak (128 bp DNA amplicon) are shown for each diagnostic group. (B) Peak areas of FynT and FynB in each electropherogram were converted to gene transcript levels and used to determine ratio of FynT to FynB expression, depicted as dot plots of CTRL (n = 14, red), AD (n = 13, blue), DLB (n = 18, amber), and PDD (n = 19, green) values with horizontal lines in each group indicating median and interquartile range. *p < 0.05, **p < 0.01, ***p < 0.001, significantly different from CTRL by Kruskal–Wallis ANOVA with *post hoc* Dunn's tests

We have previously reported specific increases of FynT immunoreactivities which contrasted with unchanged brain‐predominant FynB in AD (26). Here, using a high‐throughput gene profiling (HTA) platform, we found similar FynT upregulation in a separate cohort of AD and further extended this observation to DLB and PDD. Figure 1 shows Gene View plot where probeset PSR06025242.hg.1 (indicated by blue asterisk) corresponding to sequence for FynT‐specific exon was significantly upregulated in AD (~3 fold), DLB (~2.5 fold), and PDD (~1.8 fold) prefrontal cortex compared with CTRL. In contrast, probesets corresponding to common exons and FynB‐specific exons (yellow boxes in FynB transcript, Figure 1) showed no significant induction in the dementia subgroups. To confirm these findings, we determined the proportional amounts of FynB and FynT in two brain regions (BA9 and BA21) of aged CTRL and dementia subgroups using common primers spanning the alternative spliced exon of Fyn for RT‐PCR followed by capillary electrophoresis. The representative electropherograms in Figure 2A indicated that the FynT peaks were about 10 times lower than FynB peaks in CTRL, while elevations of FynT peaks relative to FynB were detected in the dementia subgroups. Indeed, AD, DLB, and PDD all had significantly higher FynT to FynB ratios compared to CTRL in both BA9 and BA21 (Figure 2B), with the ratios showing a high degree of correlation between the two brain regions (Pearson's *r* = 0.79, *p <* 0.001).

### 3.3 FynT upregulation correlated with cognitive impairment and neuropathological features in AD and LBD

**FIGURE 3:**
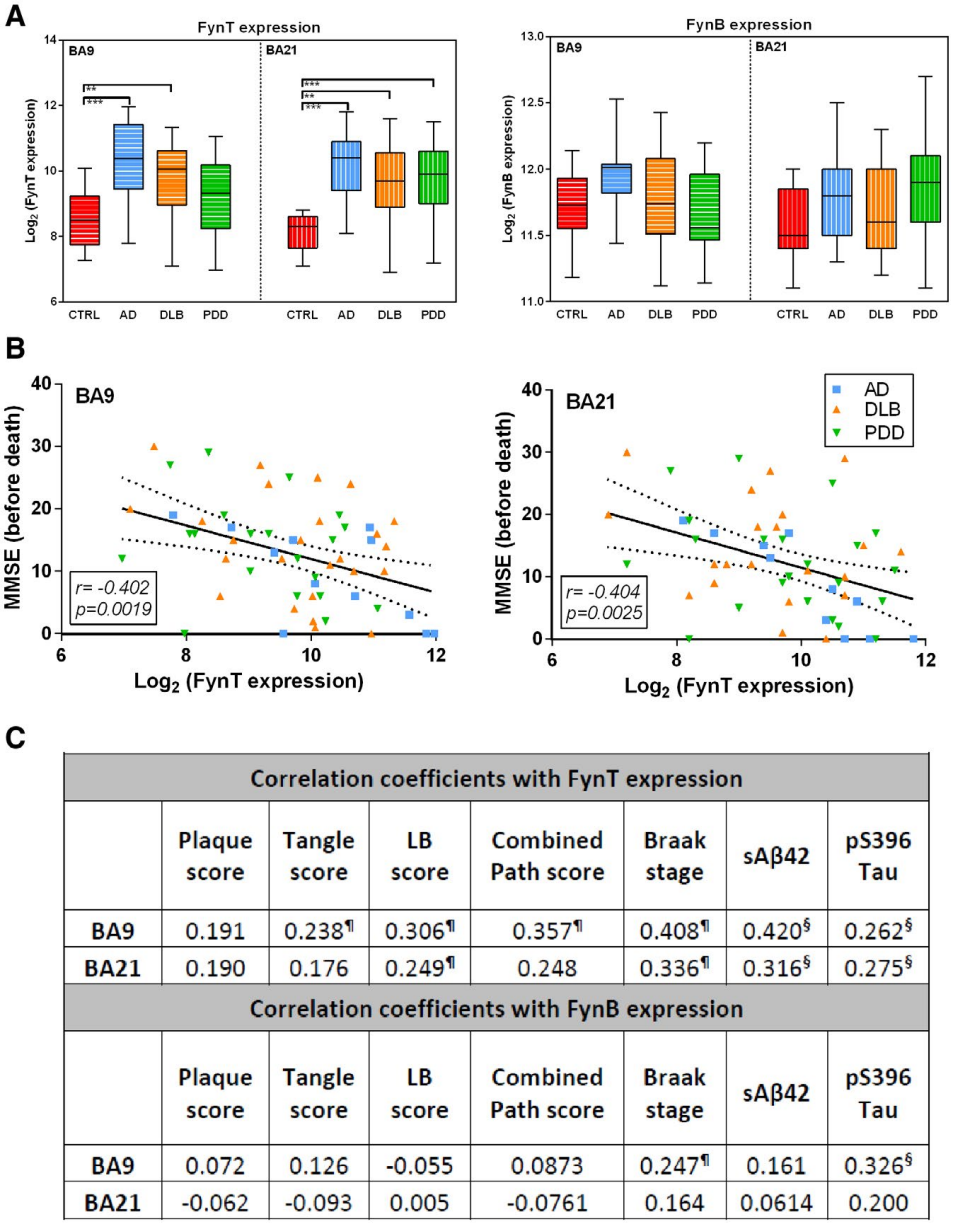
FynT upregulation in AD and LBD correlates with cognitive impairment and neuropathological features. (A) FynT and FynB expression were determined by real‐time RT‐PCR in BA9 and BA21, derived from CTRL (n = 13–15, red), AD (n = 11–12, blue), DLB (n = 25–31, amber), and PDD (n = 14–21, green). Data were Log_2_ transformed and depicted as box plots, with the horizontal intersecting lines and borders indicating median and interquartile range for each group, while whiskers show the highest and lowest values not considered outliers. (B) Scatter plots of Log_2_‐transforemd FynT expression against pre‐death cognition (MMSE) scores in BA9 (left) and BA21 (right) in the combined dementia cohort. Solid lines indicated linear regressed best‐fit curves while dashed lines indicated their respective 95% confidence intervals, with insets showing Pearson's correlation coefficients (r) and their respective *p* values. (C) Table of correlation coefficients for FynT or FynB expression with neuropathologic scores, Braak stage and biochemical measures of pS396Tau and soluble Aβ42 (in pg/mg protein) were analyzed using samples derived from CTRL, AD, and LBD. **p < 0.01, ***p < 0.001, significant differences compared to CTRL by one‐way ANOVA with *post hoc* Bonferroni's tests. ^¶^Pearson's (r) and ^§^Spearman's (ρ) correlation coefficients with *p* < 0.05

In order to investigate potential associations between Fyn perturbations and clinical or neuropathologic features, FynT and FynB expression levels were determined by real‐time RT‐PCR and correlated with pre‐death MMSE, neuropathologic scores as well as sAβ42 and pS396 Tau concentrations. Consistently, FynT was observed to be upregulated in the dementia subgroups, reaching statistical significance for AD and DLB (with a trend toward increase for PDD) in BA9, and for all three dementia subgroups in BA21 (Figure 3A left panel). We also confirmed that FynB expression were not significantly altered in the dementia subgroups (Figure 3A right panel).

Figure 3B shows that FynT expression in both BA9 and BA21 negatively correlated with pre‐death MMSE scores in the combined dementia cohort, suggesting that FynT upregulation may be associated with more severe cognitive impairment. Furthermore, Figure 3C lists the correlation coefficients between Fyn expression and (i) semiquantitative scores of amyloid plaques, neurofibrillary tangles (NFT), Lewy bodies (LB), and combined neuropathologic scores (which we have previously shown to be variably increased in LBD (18)); (ii) Braak staging and (iii) concentrations of soluble Aβ (sAβ42) and phosphorylated Tau (pS396 Tau). Interestingly, FynT expression was widely correlated with neuropathological scores (NFT, LB, and combined scores), Braak stage, sAβ42 and pS396 Tau measures, especially in BA9, while FynB did not correlate with neuropathologic scores and biochemical measures except for Braak stage and pS396 Tau in BA9 (Figure 3C). Taken together, our data suggest that FynT isoform‐specific upregulation in the neocortex may be associated with clinical severity and neuropathological burden in AD and LBD.

### 3.4 FynT upregulation correlated with markers of neuroinflammation in AD and LBD

**FIGURE 4:**
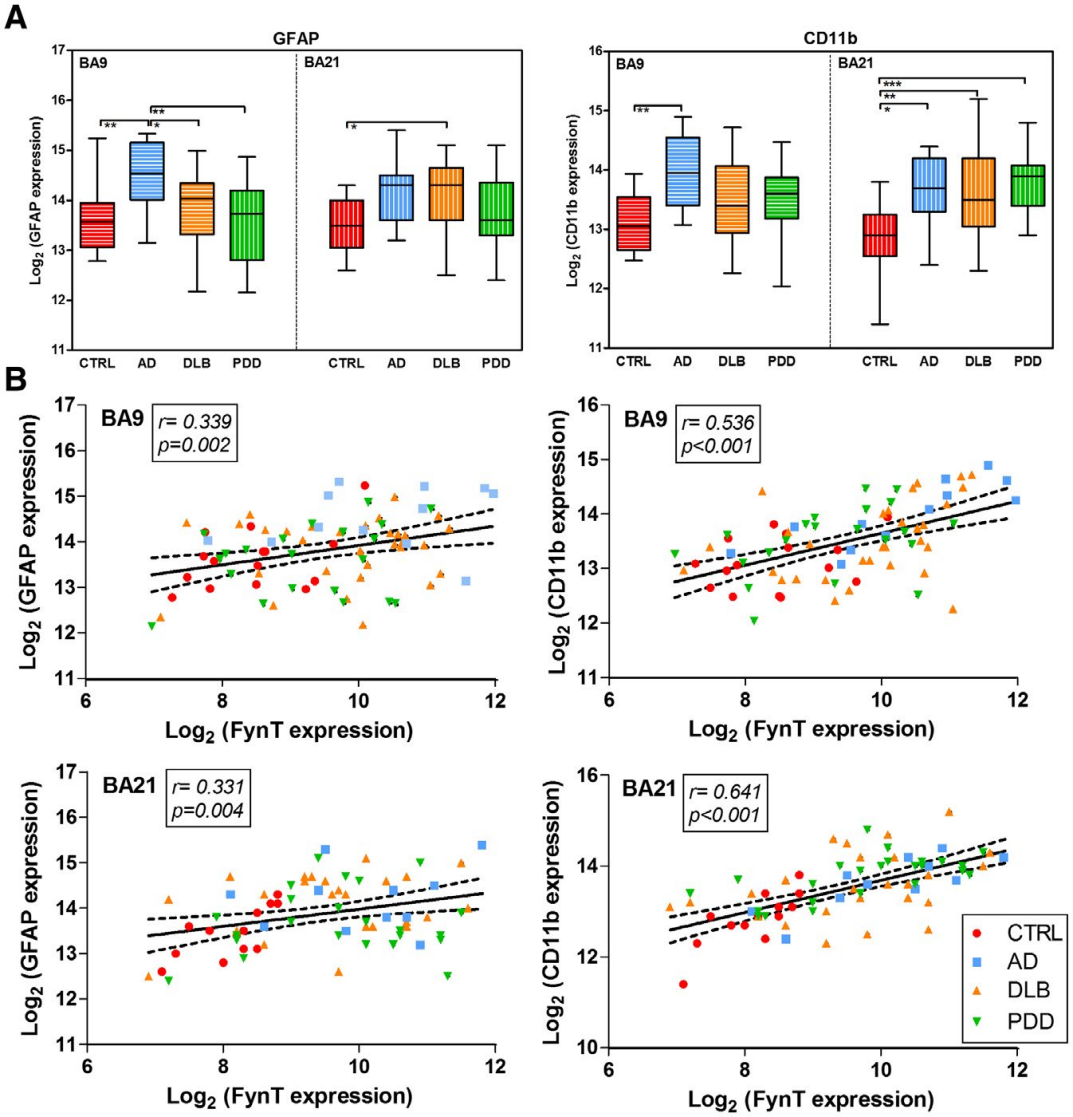
Association of FynT expression with neuroinflammatory markers in CTRL, AD, and LBD. (A) Gene expression of GFAP astrocytic marker (left) and CD11b microglial marker (right) were determined by real‐time RT‐PCR in BA9 and BA21, derived from CTRL (n = 13–15, red), AD (n = 11–12, blue), DLB (n = 25–31, amber), and PDD (n = 14–21, green). Data were Log_2_ transformed and depicted as box plots, with the horizontal intersecting lines and borders indicating median and interquartile range for each group, while whiskers show the highest and lowest values not considered outliers. (B) Scatter plots of Log_2_‐transforemd FynT expression against GFAP (left) and CD11b (right) in BA9 (top) and BA21 (bottom) of the combined cohort. Solid lines indicated linear regressed best‐fit curves while dashed lines indicated their respective 95% confidence intervals, with insets showing Pearson's correlation coefficients (r) and their respective *p* values. *p < 0.05, **p < 0.01, ***p < 0.001, significant pairwise differences between diagnostic groups by one‐way ANOVA with *post hoc* Bonferroni's tests

We have previously found that FynT induction was associated with astrocyte activation under neuroinflammatory conditions (27). In PD, microglial activation was also reported to be dependent on Fyn kinase activity (36). Here, we investigated potential associations between Fyn expression and neuroinflammatory responses using glial fibrillary acidic protein (GFAP) and CD11b expression to indicate astrogliosis and microglial activation, respectively (37, 38). Figure 4A shows significant increases of GFAP expression in BA9 for AD, and in BA21 for DLB, while expression remained unchanged for PDD in both regions (left panel). For microglia marker CD11b, expression was significantly increased in BA9 for AD, and in BA21 for all dementia subgroups (right panel). Figure 4B indicates that FynT expression correlated with both GFAP and CD11b in both brain regions in the combined cohort. To study potential sources of Fyn isoform expression in brain, we measured FynT to FynB ratios, as well as FynT, FynB, GFAP, and CD11b expression in isolated rat primary astrocyte, microglia, and neurons. Among the three cell types, we observed the highest expression of FynT in microglia, followed by astrocytes, with neurons showing the lowest expression (see Figure S1). Taken together, our results suggest that FynT induction has a role in astrogliosis and microglial activation in the neocortex of AD and LBD.

### 3.5 Synaptic dysfunction is a potential consequence of FynT upregulation in AD and LBD

To investigate the potential pathophysiological impact of FynT induction in neurodegenerative dementias, we utilized the high‐throughput transcriptome profiling data on BA9 of a subset of the cohort (9 CTRL, 9 AD, 12 DLB, and 12 PDD). The subset was selected based on RNA quality (RIN > 4) and matched demographic factors (gender, age, and PMI), and not on neuropathological scores or neurochemical variables. Stringent criteria (see details in methodology) were set to identify candidate genes which were correlated either positively or negatively with FynT. In summary, 515 FynT‐positively correlated genes and 1437 FynT‐negatively correlated genes were uncovered and independently analyzed by DAVID (34) to gain an overview of the functional profiles of associated genes in order to understand the underlying processes. Table 2 summarizes the top five GO (gene ontogeny) terms found in each category (the full list of GO terms and corresponding genes can be found in Table S2). In general, genes positively correlated with FynT were mainly associated with cell adhesion, whereas the negatively correlated genes were associated with neuronal function and components found in dendrites and synapses (Table 2). These results suggest that synaptic dysfunction and synaptopathology may be associated with FynT upregulation in AD and LBD.

**TABLE 2:** Top five* GO Terms under BP and CC categories for FynT positively and negatively correlated genes derived from CTRL, AD, and LBD transcriptome profiling data

GO term ID (categories)	Description (FynT positively correlated genes)	Count^a^	Fold enrichment^b^	Benjamini^c^
GO:0005913 (CC)	Cell–cell adherens junction	34	4.07	2.64E‐09
GO:0005925 (CC)	focal adhesion	37	3.66	4.69E‐09
GO:0030027 (CC)	lamellipodium	18	4.35	5.62E‐05
GO:0030175 (CC)	filopodium	12	6.54	9.64E‐05
GO:0001726 (CC)	ruffle	13	5.59	1.53E‐04
GO:0098609 (BP)*	Cell–cell adhesion	24	3.23	4.00E‐03
GO:0016569 (BP)*	covalent chromatin modification	14	4.51	1.52E‐02

GO term ID (categories)	Description (FynT negatively correlated genes)	Count^a^	Fold enrichment^b^	Benjamini^c^
GO:0043209 (CC)	myelin sheath	54	4.75	3.64E‐20
GO:0030054 (CC)	cell junction	91	2.65	1.62E‐15
GO:0014069 (CC)	postsynaptic density	50	3.64	2.66E‐13
GO:0008021 (CC)	synaptic vesicle	33	4.80	5.41E‐12
GO:0045211 (CC)	postsynaptic membrane	49	3.11	2.28E‐10
GO:0016192 (BP)	vesicle‐mediated transport	40	3.41	2.46E‐08
GO:0034220 (BP)	ion transmembrane transport	48	2.96	2.57E‐08
GO:0006886 (BP)	intracellular protein transport	52	2.85	4.34E‐08
GO:0000165 (BP)	MAPK cascade	54	2.67	6.03E‐08
GO:0016241 (BP)	regulation of macroautophagy	20	5.88	1.06E‐07

Gene ontology (GO) category: biological process (BP) and cellular component (CC).

^a^ Count represents the total number of genes correlated with FynT in the respective GO term.

^b^ Fold enrichment is used to measure the magnitude of enrichment.

^c^ *p* values adjusted for multiple testing using FDR < 0.05.

* For FynT positively correlated genes, only two BP terms were significant at FDR < 0.05.

### 3.6 Selective upregulation of FynT is associated with tauopathy and neuroinflammation in aged P301S mice

**FIGURE 5:**
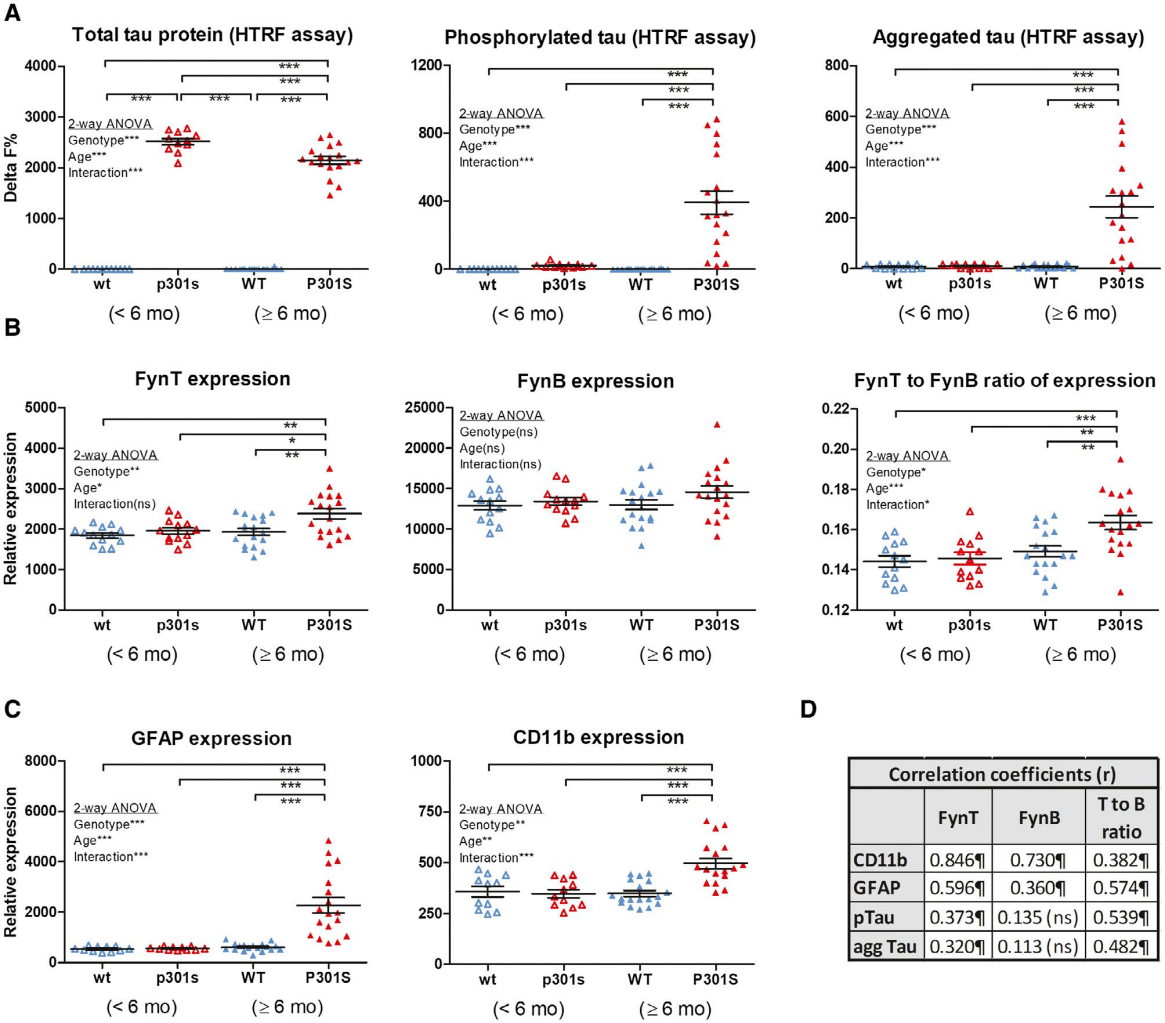
Selective upregulation of FynT in aged transgenic P301S tau mice correlates with tauopathy and neuroinflammation. (A) Determination of phosphorylated tau (pS202/T205 tau), aggregated tau and total tau protein by HTRF in the brain homogenates of P301S Tau transgenic mice and wild‐type littermate controls at age <6 mo (before onset of pathology, labels in small letters: p301 s, wt) and ≥6 mo (with disease progression, labels in capital letters: P301S, WT). RT‐PCR derived values for relative expressions of (B) FynT, FynB (together with FynT to FynB ratios) as well as (C) GFAP and CD11b in the samples as described above. Horizontal lines on the dot plots in each group indicate median and interquartile range. (D) Table of correlation coefficients for FynT, FynB, FynT:FynB with phosphorylated tau, aggregated tau, and neuroinflammation markers. *p < 0.05, **p < 0.01, ***p < 0.001, significant pairwise differences by two‐way ANOVA with *post hoc* Bonferroni's tests (Significance of the Genotype, Age, and Interaction factors are given in the respective insets of each graph). ^¶^Pearson's (r) correlation coefficients with *p* < 0.05

Finally, the potential pathophysiological links between FynT induction, tau pathology, and neuroinflammation were studied in a P301S Tau transgenic mouse model of tauopathy at two age groups: <6 mo when there is minimal brain pathology; and ≥6 mo, when the mice are known to manifest neurofibrillary tangles and neuroinflammation during disease progression. HTRF assays confirmed that P301S Tau mice expressed high levels of human tau in the brain when compared with wild‐type (WT) littermate controls at both age groups (Figure 5A, left panel). However, the younger P301S mice (<6 mo) showed minimal phosphorylated tau and aggregation, similar to WT (Figure 5A, middle and right panels). In contrast, older mice (≥ 6 mo) showed significantly increased levels of phosphorylated, aggregated tau (Figure 5A, middle and right panels). Similar to observations of human post‐mortem brains, we found significantly increased FynT expression as well as higher FynT to FynB ratio in older, but not younger, P301S mice (Figure 5B left and right panels), while FynB expression was unchanged in both age groups (Figure 5B middle panel). Furthermore, markers of neuroinflammatory responses as indicated by GFAP and CD11b expression were significantly increased only in older P301S mice (Figure 5C). Finally, Figure 5D lists the correlation coefficients between expression of Fyn isoforms, GFAP and CD11b as well as HTRF measurements of phosphorylated and aggregated Tau, and showed that both FynT and FynT:FynB correlated with the neuroinflammatory markers as well as pathologic Tau markers. Therefore, our findings suggest that selective FynT upregulation likely occurs downstream of tauopathy and is associated with disease progression, including neuroinflammation, in P301S mice.

## 4 DISCUSSION

### 4.1 FynT kinase as a potential mediator of tauopathy

While processes associated with amyloid precursor protein mismetabolism leading to accumulation of insoluble Aβ plays a major role in AD pathogenesis (39), it is the hyperphosphorylation of tau and ensuing formation of neurofibrillary tangles (NFT) which correlated more closely with disease progression, and may indeed mediate the clinical severity of AD (40, 41). As NFT (together with amyloid plaques) are a common feature of AD, PDD, and DLB, these neurodegenerative dementias can be defined as tauopathies. In this paper, we reported further evidence for a selective upregulation of the FynT isoform in AD neocortex, and extended these observations to PDD and DLB. As basal FynT expression was around one tenth of FynB expression in the normal brain (see Figure 2), we speculate that the inability of some previous studies to detect Fyn induction in AD (42, 43) was likely because of the use of non‐isoform‐specific measurements, since the amount of FynT increase would likely be masked by the unchanged, predominant FynB isoform. Importantly, we showed here that FynT upregulation was associated with astrogliosis, microglial activation, neuropathologic burden, and dementia severity, therefore, suggesting, as others have (25), that Fyn may mediate the pathophysiological link between amyloid and tau. Indeed, Fyn can regulate Aβ‐induced excitotoxicity by interacting with dendritic tau, the latter targeting Fyn postsynaptically to glutamate NMDA receptors where excitotoxic signals are transmitted (44). Aβ‐induced tau accumulation at somatodendritic compartments was also found to be Fyn‐dependent (45). In another study, binding of Aβ oligomers to postsynaptic prion proteins was shown to trigger Fyn‐mediated signaling cascades leading to tauopathy and synaptotoxicity (46, 47). Interestingly, more recent studies suggest that Fyn can also be a key regulator of tau‐associated pathology in the absence of Aβ (48, 49, 50). Conversely, tau seem to directly regulate Fyn targeting to (44); and nanodomain organization (51) within, neuronal dendrites. The complex interactions between Fyn and tau have been further demonstrated by tau's induction of Fyn autophosphorylation and kinase activity (52), contrasting with Fyn‐mediated direct (on tyrosine^18^) (53) and indirect (via CDK5 and GSK3) (54, 55) phosphorylation of tau. In summary, Fyn seems to be able to signal to tau and regulate tau‐associated pathology in both Aβ‐dependent and ‐independent pathways. Our own observations of FynT upregulation and correlations with tauopathy in AD and LBD, as well as FynT changes in P301S mice which do not manifest Aβ burden, suggest that Fyn interacts with tau in these conditions in an isoform‐specific manner, and that at least some of the pathogenic mechanisms mentioned above may be mediated by altered regulation of alternative splicing which favors FynT.

### 4.2 Potential mechanisms of FynT‐associated neuronal dysfunction and neuroinflammation in AD and LBD

Several studies have demonstrated that Fyn kinase can modulate synaptic plasticity and learning (56, 57, 58, 59). In this study, we speculated that the detrimental consequences of FynT upregulation during the dementia progression could be associated with synaptic dysfunction, based on the consistent negative correlations of FynT with genes coding for functional components of neurons and synapses (Table 2), as well as with pre‐death cognitive scores (Figure 3B). Recently, mutant P301L tau has been shown to promote aberrant Fyn nanoclustering in dendritic spines which likely contributes to synaptic dysfunction (51). In this study, we used a similar mutant P301S tau mice to demonstrate that age‐dependent increment of phosphorylated and aggregated tau was associated with FynT upregulation (Figure 5). Interestingly, while perturbations of neuronal networks and long‐term potentiation have been reported in young (2 month old) mice (60), our data suggest that these processes are unlikely to involve FynT changes, since FynT upregulation was not apparent until 6 months or older (Figure 5B). Instead, FynT might mediate the previously reported contributions of tau pathology to the development of chronic inflammatory responses during reactive astrogliosis (61). We have reported increased FynT immunoreactivity in hypertrophic cytoplasm of reactive astrocytes in the AD brain (27), which is corroborated here by the finding of significant correlations between FynT and GFAP expression (Figures 4B and 5D).

Additionally, Fyn induction in post‐mortem PD brains has been reported to be associated with neuroinflammatory responses resulting from microglial activation (62). Interestingly, the present *in vitro* data indicate the highest expression of FynT in microglia, followed by astrocyte, while neurons had the lowest expression (see Figure S1). As the correlation coefficient for FynT and CD11b expression was higher than those between FynT and GFAP or pS396Tau in the post‐mortem study, our data suggest FynT upregulation in the AD and LBD brain could due in large part to activation of predominantly FynT‐expressing microglia, which may in turn contribute to chronic neuroinflammation‐associated neuronal damage (63). However, given the significant (albeit weaker) correlations between FynT and GFAP, as well as previously reported localization of FynT in reactive astrocytes (26), we postulate that astrogliosis underlie at least part of the observed FynT upregulation. Here, it is worth noting that FynT likely plays a critical role in astrocyte activation, as we have previously shown that neuroinflammatory responses to tumor necrosis factor is abolished in astrocytes expressing a kinase‐dead mutant of FynT (27). Therefore, when considered in the context of previous studies, our current *in vitro* and post‐mortem data suggest that in AD and LBD, FynT upregulation likely occurs in response to tau phosphorylation and NFT formation, and mediates astrocyte and/or microglial activation, leading in turn to neuroinflammation‐associated synaptic dysfunction and neuronal damage.

### 4.3 Isoform‐specific role of FynT as therapeutic target for neurodegenerative dementias

Fyn kinase has already been proposed as a potential therapeutic target for AD (64). In this regard, a previous study showed that inhibiting Fyn in an AD mouse model restored synaptic density, limited tau aggregation, reduced microglial activation, and subsequently reversed memory deficits without attenuating Aβ load (58). Recent findings consistently demonstrated that both pharmacological Fyn inhibition and depletion of Fyn (via gene knockout) in tauopathy models resulted in behavioral improvements, recovery of synaptic loss, together with reduced tau phosphorylation and gliosis, suggesting that Fyn is an essential pathogenic factor in tauopathies (48, 49, 50). This has expanded the application of Fyn as a therapeutic target for tauopathies in addition to AD. However, it is worth noting that AZDO530, a Src family kinase inhibitor which showed promise in animal studies, demonstrated no statistically significant improvement on neuroimaging outcome in AD patients in a year‐long Phase IIa clinical trial (although a trend toward less shrinkage of the hippocampus and entorhinal cortex was observed in the treated group)(65). One insight that may be gleaned from our study is the need to refine the target to Fyn, and specifically the FynT isoform, for potentially improved efficacy as well as reduced off‐target adverse effects, since the major brain variant FynB has important physiological functions and should not be targeted. Furthermore, when a FynT‐specific compound does become available, our data suggest extending assessments of their clinical utility to LBD in addition to AD.

### 4.4 Study limitations

There are several limitations apparent in this study which are related to the limited number of available samples. Therefore, in interpreting findings where changes are found in one, but not both of the brain regions examined (e.g., significant correlation between FynT and neurofibrillary tangle (NFT) scores in BA9 prefrontal cortex but not BA21 temporal cortex, see Figure 3C), it is unclear whether the observed regional differences has a genuine pathophysiological basis (for, e.g., more active disease process in BA9 versus widespread cell loss in BA21(66)) or was because of higher data variability in BA21 resulting in nonsignificance of the association. On the contrary, FynT expression seemed to be increased in both regions in LBD, but did not reach statistical significance in BA9 for PDD (Figure 3A). While higher AP and NFT loads are detected in DLB compared with PDD (12, 67), and NFT (which we have shown here to be strongly correlated with FynT) is known to originate from the entorhinal, hippocampal, and neocortical regions of the temporal lobe followed by progression into the prefrontal and other neocortical areas (68), we cannot at present confirm whether these regional differences are DLB‐ or PDD‐specific because of associations with pathological burden, necessitating follow‐up studies on larger cohorts looking at multiple brain regions.

Second, the current study has not studied Fyn associations with LBD‐specific lesions (α‐synuclein‐containing Lewy bodies, LB) in detail. While the focus of our study has been on AD pathological features which are present in both AD and LBD, FynT may also be linked to LB, as suggested by Figure 3C. However, it is at present unclear whether, or how, FynT may be related to α‐synuclein aggregation and related pathophysiology *per se*, as assays of soluble α‐synuclein revealed unchanged baseline cortical concentrations across controls and dementia subgroups in agreement with previous studies (69, 70) which did not correlate with Fyn or other neuropathological measures (data not shown). These negative findings suggest that other potentially pathogenic species of α‐synuclein, for example, Ser129‐phosphorylated α‐synuclein (71) need to be measured in follow‐up studies of potential Fyn involvement in LBD‐specific pathology.

Finally, the current study employed a genomics approach with the use of microarrays and RT‐PCR to study Fyn expression and associations with a wide range of gene expression changes. There is therefore a lack of histological and cellular localization data, which is mitigated somewhat by (i) the use of primary cultures to provide *in vitro* validation of the likely sources of FynT, namely, astrocytes and microglia (Figure S1); and (ii) our previous study which showed the localization of FynT in activated astrocytes in AD (26). However, follow‐up studies are needed to confirm the findings for microglia and also extend them to LBD.

## 5 CONCLUSIONS

Using microarray platforms, we report FynT isoform‐specific upregulation in the neocortex of AD, DLB, and PDD. The observed induction of FynT was closely associated with tauopathy and neuroinflammation, and may mediate their deleterious effects on synaptic and cognitive functions. In corroboration, age‐dependent FynT upregulation was detected in P301S mutant tau transgenic mice, which was significantly correlated with phosphorylated and aggregated tau, as well as with markers of microglial and astrocytic activation. Our findings point to FynT as the pathogenic Fyn isoform for AD and LBD, and propose a refinement of therapeutic approach from nonspecific inhibition of Src family kinases to a more focused search for compounds which specifically target FynT as potential treatment for AD‐related chronic neuroinflammation.

## AUTHOR CONTRIBUTIONS

MGKT and MKPL conceived the study and designed the project; CYBL, JHL, FTWL, and CL performed the experiments; CB and PTF provided post‐mortem and clinical data; CYBL, JHL, and MGKT analyzed the data; MGKT and MKPL wrote the first draft. All authors have read and approved the manuscript.

## CONFLICT OF INTEREST

The authors declare that they have no conflict of interest.

## CONSENT FOR PUBLICATION

All authors gave consent for publication.

## ETHICS APPROVAL AND CONSENT TO PARTICIPATE

For the post‐mortem study, ethics approval for the collection and study of brain tissues received Institutional Review Board approval in both the United Kingdom (08/H1010/4) and Singapore institutions (NUS 12‐062E), and informed consent was obtained from participants’ next‐of‐kin prior to removal of brain. The mice studies were approved by the SingHealth Group Institutional Animal Care and Use Committee (2017/SHS/1280).

## Data Availability

The human transcriptome data set has been deposited in Gene Expression Omnibus (GEO) data repository (Access #GSE150696). Other data that support the findings of this study are available on reasonable request from the corresponding authors.
