# Supplementary material for: Isoform‐specific upregulation of FynT kinase expression is associated with tauopathy and glial activation in Alzheimer's disease and Lewy body dementias
Source: Brain Pathol. 2021 Jan 29;31(2):253–66. doi: 10.1111/bpa.12917 (PMC8017997; doi:10.1111/bpa.12917)
Supplement: Supplementary file 1 [file BPA-31-253-s002.pdf]

Low *et al.* Isoform-specific upregulation of FynT kinase expression is associated with tauopathy and glial activation in Alzheimer's disease and Lewy body dementias

Supplementary Fig. S1

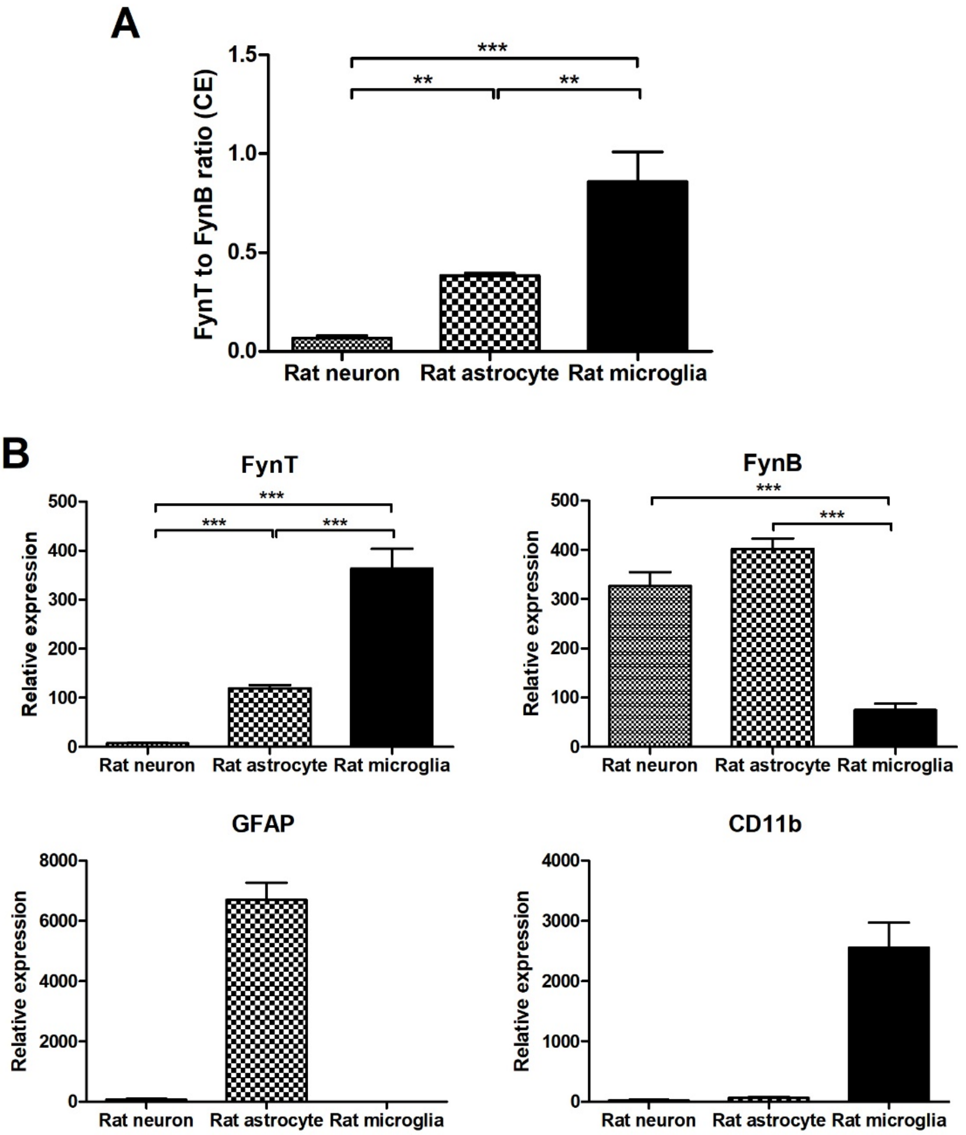

Figure S1. Higher proportions of FynT expression in rat primary microglia cells. A. Endogenous FynT to FynB ratios were determined by RT-PCR followed by CE (see Methods) in rat primary neurons, astrocytes and microglia cells. Data are mean ± S.E.M of n=3-6 independent isolations of each primary culture. B. FynT and FynB, GFAP and CD11b expression were determined by real-time RT-PCR in primary neurons (n=6), astrocytes (n=11) and microglia cells (n=5).
**p<0.01, ***p<0.001, significant pairwise differences between diagnostic groups by one-way ANOVA with *post-hoc* Bonferroni's tests.
