# Supplementary material for: Isoform‐specific upregulation of FynT kinase expression is associated with tauopathy and glial activation in Alzheimer's disease and Lewy body dementias
Source: Brain Pathol. 2021 Jan 29;31(2):253–66. doi: 10.1111/bpa.12917 (PMC8017997; doi:10.1111/bpa.12917)
Supplement: Supplementary file 2 [file BPA-31-253-s001.pdf]

Low *et al.* Isoform-specific upregulation of FynT kinase expression is associated with tauopathy and glial activation in Alzheimer's disease and Lewy body dementias

Supplementary Table S1. Primers used in real-time PCR reactions

Primer Name	Forward primer seq (5'-3')	Reverse primer seq (5'-3')	Accession no. (product size)
Hs_FynB	CTGCTGCCGCCTAGTAGTTC	GTGTTTCCATTCCAGGTACC	NM_002037(168bp)
Hs_FynT	CATCGAGTTGTACCCCACAA	GTGTTTCCATTCCAGGTACC	NM_153047(136bp)
Hs_GFAP	GGGAGCTTGATTCTCAGCAC	AATTGCCTCCTCCTCCATCT	NM_002055 (178bp)
Hs_CD11b	GCCGGTGAAATATGCTGTCT	GCGGTCCCATATGACAGTCT	NM_001145808(199bp)
Hs_18S rRNA	CCTGCGGCTTAATTTGACTC	CGCTGAGCCAGTCAGTGTAG	M10098(310bp)
Hs_β-actin	ACTGGAACGGTGAAGGTGAC	AGAGAAGTGGGGTGGCTTTT	NM_001101(169bp)
Hs_GAPDH	TGACATCAAGAAGGTGGTGAAG	TTACTCCTTGGAGGCCATGTG	M33197(241bp)
Ms FynB	CTGCTGCCGCCTAGTAGTTC	GTATTTCCATTCCAGGTACC	NM_001122893(168bp)
Ms FynT	CATCAAGTTGTACCCCACAA	GTATTTCCATTCCAGGTACC	NM_008054(136bp)
Ms GFAP	TGAGGCAGAAGCTCCAAGAT	CACGTGGACCTGCTGTTG	NM_001131020(215bp)
Ms CD11b	CAGCATCAGTACCAGTTCAACA	CTGCAACAGAGCAGTTCAGC	NM_001082960 (223bp)
Ms 18S rRNA	CCTGCGGCTTAATTTGACTC	CGCTGAGCCAGTCAGTGTAG	NR_003278(319bp)
Ms GAPDH	GGCATTGCTCTCAATGACAA	TGTGAGGGAGATGCTCAGTG	NM_008084(200bp)
Ms β-actin	ACTGGAACGGTGAAGGCGAC	GAGGGTGAGGGACTTCCTGT	NM_007393 (175bp)
Fyn	GGCCCAGTTTGAGACCCTTC	*GTGTTTCCATTCCAGGTACC	(228bp for FynB)<br>(219bp for FynT)
P301S	GGCATCTCAGCAATGTCTCC	GGTATTAGCCTATGGGGGACAC	P301S genotype(450bp)
TCRD	CAAATGTTGCTTGTCTGGTG	GTCAGTCGAGTGCACAGTTT	Control (200bp)

Hs = Human; Ms = Mouse
*Indicates Fyn primer sets for fragment analysis using capillary electrophoresis, with 5' labeled 6-FAM on reverse primer
